# Scleral bridging technique for preventing PreserFlo microshunt exposure: A case report

**DOI:** 10.1097/MD.0000000000038847

**Published:** 2024-07-05

**Authors:** Shunsuke Nakakura, Yuki Nagata, Yasuko Fujisawa, Yoshiaki Kiuchi

**Affiliations:** a Department of Ophthalmology, Saneikai Tsukazaki Hospital, Himeji, Japan; b Department of Ophthalmology and Visual Sciences, Graduate School of Biomedical Sciences, Hiroshima University, Hiroshima, Japan.

**Keywords:** glaucoma, PreserFlo microshunt, sclera, shunt exposure, tube exposure

## Abstract

**Introduction:**

The use of the PreserFlo microshunt is gaining popularity owing to its ease of implantation and reduced need for postoperative intervention compared to conventional trabeculectomy.

**Patient concerns:**

However, microshunt exposure remains a severe complication of PreserFlo surgery, particularly in patients with a thin Tenon capsule and conjunctiva. However, the actual thickness and intensity of the Tenon capsule or conjunctiva can be confirmed only during surgery.

**Diagnosis:**

Exfoliation glaucoma with previous several glaucoma surgeries with thinner Tenon capsule or conjunctiva.

**Interventions:**

We performed PreserFlo implantation with a surgical technique to recover a thin Tenon capsule and conjunctiva by creating a half-thickness rectangular scleral flap under the shunt and covering it over the microshunt until the distal part, similar to the bridge.

**Outcomes:**

The patient had better intraocular pressure control with positive cosmetic appearance using this technique.

**Conclusion:**

This technique will be beneficial for both preventing exposure and holding down the top, in addition to improving cosmetic appearance.

## 1. Introduction

Minimally invasive glaucoma surgeries and microinvasive bleb surgery have gained popularity worldwide as alternatives to conventional trabeculectomy.^[1–4]^ Although the PreserFlo microshunt (Santen Pharmaceutical Co., Osaka, Japan) has been shown to more strongly reduce intraocular pressure (IOP) as compared with XEN,^[5,6]^ its outcomes remain inferior to those of trabeculectomy.^[7,8]^ However, as compared with trabeculectomy, PreserFlo may result in fewer postoperative complications,^[8]^ require a less steep surgical learning curve,^[9,10]^ and bridge the gap between microinvasive glaucoma surgery and traditional filtering surgery.^[11]^ Among the complications of PreserFlo, its exposure over the conjunctiva is a severe complication that increases the risk of foreign-body sensation and infection.^[12–15]^ Reported potential risks in patients’ backgrounds for microshunt exposure are the lack of Tenon capsule,^[12,13]^ blepharitis,^[12]^ loose Nylon suture,^[14]^ and a history of multiple surgeries.^[12–15]^ Intact conjunctiva and Tenon capsule are positive indications for PreserFlo microshunt surgery; however, the actual thickness and intensity of Tenon capsule or conjunctiva can be confirmed only during the surgery.

Here, we report a technique for surgical recovery that prevents microshunt exposure in patients with thinner Tenon capsule and conjunctiva.

## 2. Case and technique presentation

The Institutional Review Board of Saneikai Tsukazaki Hospital approved the study (No. 221050), and the research was performed in accordance with the Declaration of Helsinki.

A 72-year-old man with exfoliation glaucoma was referred to our hospital because of increasing IOP in the right eye despite a previous trabeculectomy and 2 bleb revisions. At the first visit, the patient’s IOP was 28 mm Hg on the right side with topical latanoprost 0.005%/carteolol 2% fixed combination and brinzolamide 1% and 15 mm Hg on the left side without medication. His best-corrected visual acuity (logMAR) was 1.0 on the right and −0.17 on the left. The temporal superior conjunctiva was scarred from the previous surgery; therefore, we scheduled a PreserFlo microshunt in the inferior temporal quadrant.

The patient has provided informed consent for publication of the case.

## 3. Surgical technique

**Figure 1.**
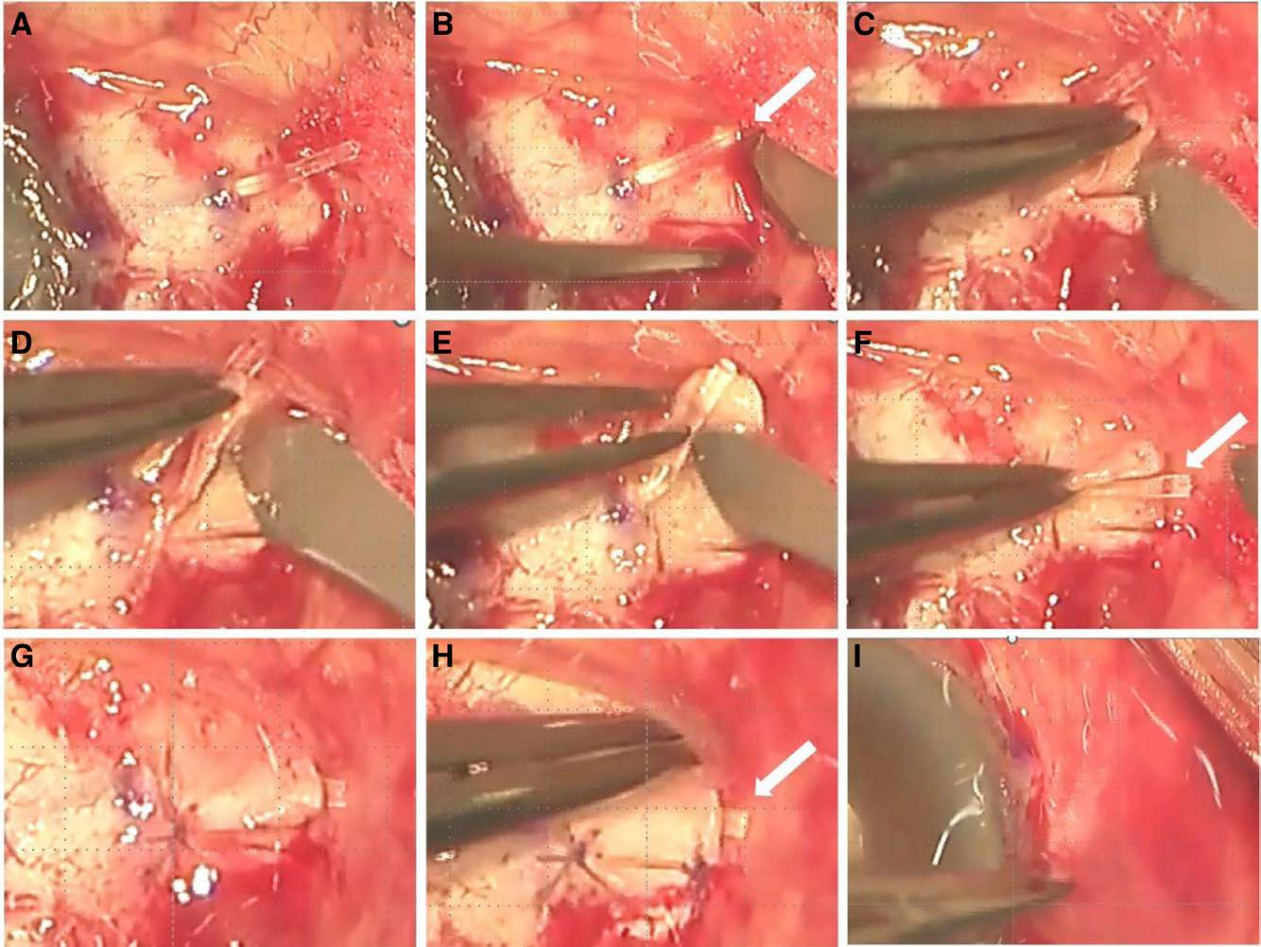
Scleral bridging surgical technique.

**Figure 2.**
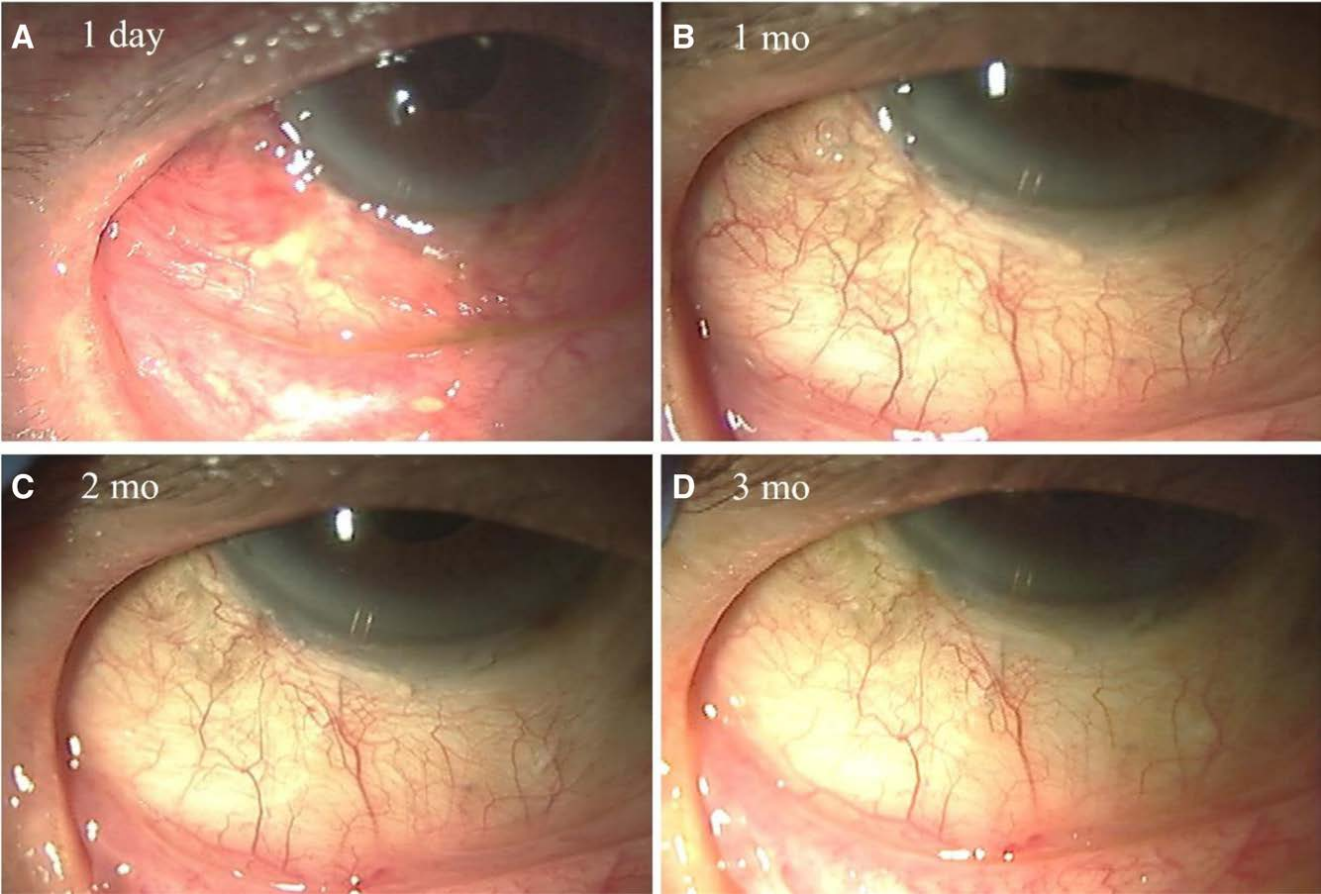
Slit-lamp photography after surgery.

After a 7-mm limbal 4-mm radial conjunctival incision, we achieved sub-Tenon anesthesia and hemostasis by applying 2% lidocaine with epinephrine. Using a neurosurgical pad, 0.04% mitomycin C was applied for 3 minutes under the conjunctiva at the distal space of the temporal inferior, followed by rinsing with approximately 50 mL saline solution. At this time, we confirmed a very thin conjunctiva with almost no Tenon capsule. We created a 1-mm side port in the cornea. Using a dedicated knife, an incision was made 3.5 mm from the limbus to ensure that the knife tip entered the anterior chamber. We inserted the PreserFlo and injected a balanced salt solution from the port to confirm the flow from the PreserFlo. When attempting to suture Tenon capsule and the conjunctiva over PreserFlo using 8-0 Vicryl, we could not pull the Tenon capsule forward. In addition, because of the thinner conjunctiva, we performed the scleral bridging technique to prevent PreserFlo microshunting. Figure 1 shows the surgical techniques used in this study. After inserting the PreserFlo microshunt (Fig. 1A), a half-thickness scleral flap (a rectangle of approximately 3 mm × 3 mm) was created using a blade knife (Fig. 1B–E). We designed the scleral incision to barely show the distal top of the PreserFlo not to bury under the scleral flap (white arrow in Fig. 1). After overlapping the PreserFlo (Fig. 1F), the scleral flap was sutured softly like a bridge over the PreserFlo (Fig. 1G and H) using 8-0 Vicryl. Finally, the conjunctiva was sutured to the corneal limbs using an 8-0 Vicryl (Fig. 1I). The patient’s IOP was 11, 10, 13, and 16 mm Hg at 1 day, 1 month, 2 months, and 3 months, respectively, without topical glaucoma medication. Figure 2 shows the conjunctival appearance after surgery. Figure 2A shows the edematous conjunctiva 1 day after surgery. As shown in Figure 2B–D, the PreserFlo was almost hidden under the scleral flap with good cosmetic appearance.

## 4. Discussion

Scleral bridging is an easy and quick technique for use during PreserFlo surgery and is especially useful for patients with thinner conjunctiva and thinner or lack of Tenon capsule. We believe that this technique has the following benefits: it is easy to operate because the flap thickness and size are freely designed; the scleral flap that covers PreserFlo can suppress the rise of the top because it is useful for preventing microshunt exposure; and unlike Tutoplast,^[16]^ donor sclera, and donor cornea, there is no concern about cosmetic or cost problems. Additionally, this technique will be easier than previously reported autologous scleral graft techniques for glaucoma drainage device.^[17,18]^

Bunod et al^[12]^ reported 2 cases of PreserFlo exposure, which were ultimately removed after revisions. Michaels et al^[13]^ reported 1 case of PreserFlo exposure that was finally removed after revision. Fahy et al^[14]^ reported 1 case of PreserFlo exposure but recovered it by repositioning to the temporal area. In the single case reported by Durr et al,^[15]^ no information was available after exposure. Therefore, once the exposure occurred, removal of PreserFlo was highly frequent.

## 5. Conclusion

The scleral bridging technique will be beneficial for both preventing exposure and holding down the top, adding to the positive cosmetic appearance.

## Author contributions

**Conceptualization:** Shunsuke Nakakura, Yuki Nagata, Yoshiaki Kiuchi.

**Data curation:** Shunsuke Nakakura, Yuki Nagata, Yasuko Fujisawa, Yoshiaki Kiuchi.

**Investigation:** Shunsuke Nakakura.

**Validation:** Shunsuke Nakakura, Yasuko Fujisawa, Yoshiaki Kiuchi.

**Visualization:** Shunsuke Nakakura, Yuki Nagata, Yoshiaki Kiuchi.

**Writing—original draft:** Shunsuke Nakakura, Yoshiaki Kiuchi.

**Writing—review & editing:** Shunsuke Nakakura, Yoshiaki Kiuchi.

**Formal analysis:** Yuki Nagata.

**Supervision:** Yasuko Fujisawa, Yoshiaki Kiuchi.
